# Anticancer potential of *Carica papaya Linn* black seed extract against human colon cancer cell line: in vitro study

**DOI:** 10.1186/s12906-023-04085-7

**Published:** 2023-07-29

**Authors:** Nadia S. Mahrous, Enas A. Noseer

**Affiliations:** 1 Department of Zoology, Faculty of Science, South Valley University, Qena, 83523 Egypt; 2 Department of Biochemistry, Faculty of Veterinary medicine, Aswan University, Aswan, 81528 Egypt

**Keywords:** Carica papaya Linn, Colorectal cancer, Caco-2, Apoptosis, Gene expression

## Abstract

**Background:**

Since cancer is one of the most prevalent diseases in the world, further studies are needed to identify the effective therapeutic modalities. The second deadliest and third most common cancer is colorectal cancer (CRC). Papaya (*Carica papaya* Linn) seeds offer anti-cancer properties that can cure various types of cancer, such as liver and prostate cancer.

**Methods:**

The study aimed to evaluate the anti-cancer activity of *Carica papaya* seed extract on colorectal cancer cell lines (Caco-2) and used techniques to assess the anti-cancer potential. The effectiveness of SE on cell proliferation and the viability of HTB-37 Caco-2 and C-166 cells were assessed using the MTT test. Real-time RT-PCR was used to measure gene expression levels and evaluate the activity of genes involved in apoptosis, including *caspase-3, p53, Cycs*, and *Bcl-2*. Finally, flow cytometry was used to analyze apoptosis induction by detecting changes in cell morphology and DNA content.

**Results:**

The study showed that the MTT reduction assay was dependent on cancer cell type and concentration of SE compared to the control cells and C-166, with a mean IC_50_ value of 9.734 ug/ml. The cytotoxicity was accompanied by some morphological alterations in the colorectal cancer cell line (Caco-2). The expression of the genes for *p53*, *Cycs*, and *caspase-3* was substantially up-regulated, while *Bcl-2* was dramatically down-regulated compared to control cells. The cell cycle arrested at the G2-M phase and the presence of early and late apoptotic characteristics post-treatment increased the apoptotic profile.

**Conclusion:**

It concluded that papaya seeds aqueous extract could act as a novel therapeutic option for colorectal cancer (CRC).

## Background

The tropical fruit papaya (*Carica papaya*) is cultivated for its leaves, seeds, and roots, which are widely used in traditional medicine to treat a wide range of illnesses [1]. Papaya is revered as a miraculous fruit because it contains many vitamins, including A, B, and C, monounsaturated fatty acids, proteins, carbs, and high alkaloids like carpaine and pseudocarpaine, and other bioactive compounds. Papaya seeds have more medical advantages than papaya flesh [2]. The roots, leaves, fruits, latex, juice, and seeds of the papaya plant have all been found to contain phytochemicals [3].

Numerous components in papaya have anti-cancer effects [4]. Papaya has been linked to chemo-preventive properties, including activating tumor-suppressor genes, deactivating oncogene products transcriptionally, and minimizing oxidative damage by acting as free radical scavengers [5].

The papaya seed and pulp contain benzyl glucosinolate, which is broken down by the enzyme myrosinase to create benzyl isothiocyanate, which has anticancer effects. It has been demonstrated to stop the expansion of specific cancer cell lines [6]. Isocyanate chemicals enhance their anticancer activities through cytoprotection, antioxidative, anti-inflammatory, and genoprotective mechanisms [7]. Second, papaya fruits are rich in lycopene, which was found to be effective in killing liver cancer cells [8]. Papaya seed extract showed anticancer effects in acute promyelocytic leukaemia. Furthermore, papaya seeds’ capacity to successfully inhibit the growth of prostate cancer cells raises the possibility that they possess anticancer properties [9]. In addition to limiting the responses of solid cancer cells in the cervix, breast, liver, lung, and pancreas, papaya leaves also have an antitumor effect [10].

Colorectal cancer (CRC) is the second most prevalent rectal cancer [11] and is becoming more widespread in developing countries. The CRC is a diverse set of disorders that are brought on by several mutations and mutagens. The body’s immune system can react to specific dietary and lifestyle patterns, promoting an adenoma to grow and transform into cancer [12].

One of the best methods of preventing colorectal cancer is using non-steroidal anti-inflammatory medicines (NSAIDs). Three of the key anticancer effects of NSAIDs are the reduction of tumor cell growth, angiogenesis, and apoptosis, as well as the disruption of the inflammatory environment. However, NSAID use over an extended period has been linked to poor clinical outcomes [13].

Natural remedies derived from medicinal plants can enhance health [14]. The use of immunomodulation medications, including phytochemicals has developed as a safe and effective alternative to herbal remedies, which are used for both the prevention and treatment of cancer. Herbal treatments are thought of as a complimentary way to contemporary medicine [15].

The *Bcl-2* gene is the most well-known member of the gene family that regulates cell homeostasis. Furthermore, whereas certain Bcl-2 family members induce apoptosis, others inhibit it [6]. As a tumor suppressor gene and apoptosis regulator, it is important to note that *p53* interacts with pro-apoptotic members of the Bcl-2 family, activates caspase, and eventually participates in the intrinsic apoptosis pathway [16]. Procaspase-9, cytochrome C, and apoptotic protease activating factor (Apaf-1) are released from the mitochondria’s interior and enter the cytosol to form the apoptosome complex. The apoptosome converts procapsase 9, which promotes caspase-3 activation. Active caspase-3 eventually encourages apoptosis.

## Materials and methods

### Cell lines

Human colorectal adenocarcinoma cell lines Caco-2 (HTB-37), and normal cell line mouse endothelial cell (C-166) were purchased from VACSERA, Giza, Egypt. RPMI-1640 medium and Trypsin solution were purchased from (Adwia-Egypt). Fetal bovine serum was procured from Gibco, USA. Ethylene diamine tetra acetic acid was obtained from Gibco, USA, and 0.05% concentration was freshly prepared. Dimethyl sulfoxide (DMSO) was purchased from (Sigma-Aldrich, USA).

### Plant material

Papaya fruit was obtained from the South Valley University, Qena, Egypt. Mature, healthy fruits were selected for their consistency in size, shape, and color. Fruits were collected in conformity with the laws and regulations that applied.

#### Preparation of SE (*Carica Papaya Linn.* seeds aqueous extract)

According to Alotaibi et al. [9], papaya seeds are harvested from fully grown, healthy fruits and dried. Fifty grams of papaya seeds were washed with distilled water, dried in the air, and then reused for preparing the extract. The grinding process was performed through liquid nitrogen, and the exfoliated powder was dissolved in 300 mL of distilled water and positioned on a metallic shaker overnight. The resultant solution was centrifuged for 1000 rpm X 20 min. The residues were removed via washing three times, and the supernatant was collected till be fully strengthened to 100%. Finally, the extract was stored at – 20 °C till used.

### Cytotoxicity (MTT) assay

The MTT proliferation assay was used to assess cell viability. MTT [3-(4,5-dimethylthiazol-2-yl)-2,5-diphenyltetrazolium bromide] test reacts in the medium by forming purple formazan crystals depending on mitochondrial reductase enzyme activity. Each Caco-2 cancer cells and C-166 spread in 75 cm^2^ cell culture flasks (TPP-Swiss) in RPMI-1640 medium supplied with 10% (v/v) fetal bovine serum and incubated in 5% CO^2^ incubator (Jouan-France) at 37 °C.

Trypsinization of cells via 0.25% (w/v) trypsin solution and 0.05% (v/v) ethylene diamine tetra acetic acid (EDTA) for 5 min. 2 × 10^5^ cells/ ml were plated in 96-well cell culture plates and incubated at 37 °C for 24 h till achieving the confluence.

Dead cells were eliminated through washing by phosphate-buffered-saline (PBS), then 50 µl of MTT solution was added to each well and incubated overnight. 50 µl of dimethyl sulfoxide (DMSO) was added per each well to solubilize the precipitates. Plates were incubated for 30 min at 38 °C then positioned in microplate reader (Biotek ELX − 800, USA) at 570 nm wavelength. The IC_50_ value (inhibitory concentration at 50%) is used to determine the specific concentration of papaya seed extracts required to reduce the population of 50% of viable cells. The IC_50_ value was calculated via Masterplex 2010 software [17].

Viability percentage (%) = Mean OD of test dilution × 100/Mean OD of control wells. The IC_50_ value was determined using GraphPad Prism software (v.6, GraphPad Software, La Jolla, CA, USA).

### Cell cycle analysis

Flow cytometry device was used to examine and analyze the cells one by one at 37 °C at 488 nm wavelengths [18].

### Annexin-V-FITC assay

The apoptotic profile was determined using an Annexin-V/PI staining assay [19]. IC_50_ of papaya seed extract dissolved in RPMI-1640 medium and added to both treated and untreated Coca-2 cells for 24 h. The cells were collected and fixed in PBS with 70% (v/v) ethanol overnight at 4 °C. The cells were then resuspended in PBS containing 40 g/ml PI and 0.1 mg/ml RNase in the dark. For adherent cells, gently trypsinize and wash cells once with serum-containing media before staining with annexin V-FITC and PI according to the manufacturer’s instructions. Finally, the fluorescence intensity of stained treated and untreated Caco-2 cancer cells was determined using Becton-Dickinson flow cytometry.

### Evaluation of apoptosis-related genes

All RNA from untreated and treated Caco-2 was extracted via the Gene JET RNA Purification kit. The spectrophotometer was run at 260/280 nm ratio for RNA elevation. The reverse transcription kit was used to produce the cDNA, which was then frozen at -80 °C until it was needed. Quantitative real-time PCR was performed on a Rotor-Gene Q cycler (Qiagen, Germany) using QuantiTect SYBR Green PCR kits (Qiagen, Germany) forward and reverse primers. The nucleic acid sequences of the primers were as follows:

**Table:** 

Primer	The nucleic acid sequences
*p53*	(F: 5’-CCCCTC CTG GCC CCT GTC ATC TTC-3’and R: 5’- GCA GCG CCT CAC AAC CTC CGT CAT-3’)
*CyCs*	(F: 5’- CCA ATG AAG ATC CCA CAT G-3’ and R: 5’-CCA GGA AAG TAG GGG TTG AAG T-3’)
*Casp3*	(F: 5’-TTC ATT ATT CAG GCC TGC CGA GG-3’ and R: 5’-TTC TGA CAG GCC ATG TCA TCC TCA-3’)
*Bcl-2*	(F: 5’- CCT GTG GAT GAC TGA GTA CC-3’ and R: 5’- GAG ACA GCC AGG AGA AAT CA-3’)
*β-acting*	(F: 5’ GTG ACA TCC ACA CCC AGA GG -3’ and R:5 – ACA GGA TGT CAA AAC TGC CC − 3’).

The initial activation occurs at 95 °C for 25 min, followed by denaturation at 94 °C for 40 cycles for 15 s, then annealing for 30 s at 62 °C and the end elongation cycle for 30 s at 70 °C. Melting curves were performed after real-time PCR to demonstrate the specific amplification of single products of interest. The amplification efficiency of the primer was determined using standard curve assay. Relative fold changes in the expression of target genes (*p53, CyCs, Casp3*, and *Bcl-2*) were calculated using the comparative 2 − ΔΔCt with an internal control gene to know the value of target genes.

### Statistical analysis

The recorded data was analyzed by one-way ANOVA using SPSS version 14 and differences from (*P < 0.05*) were considered significant.

## Results

### Cytotoxicity assay

**Fig. 1:**
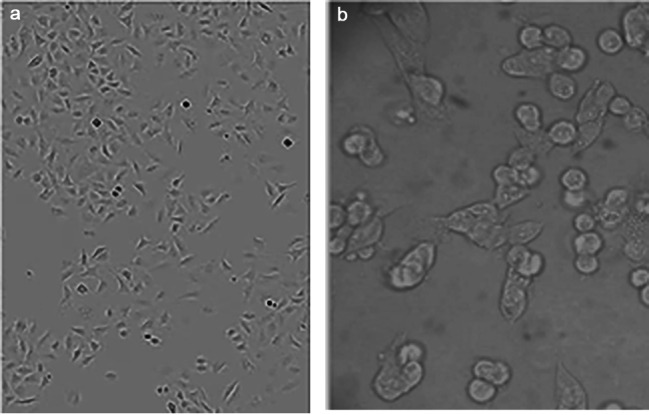
Microscopic examination of Caco-2 treated cells using the IC50 value of extracted papaya seed using inverted microscope. (**a**) untreated colerectal cancer cell show cluster and condensation of cells. (**b**) morphological changes of treated colorectal cancer cell with papya seeds extract in which the Caco2 cells became void of cells and cellular irregularities, shrinkage with condensed cell components

**Fig. 2:**
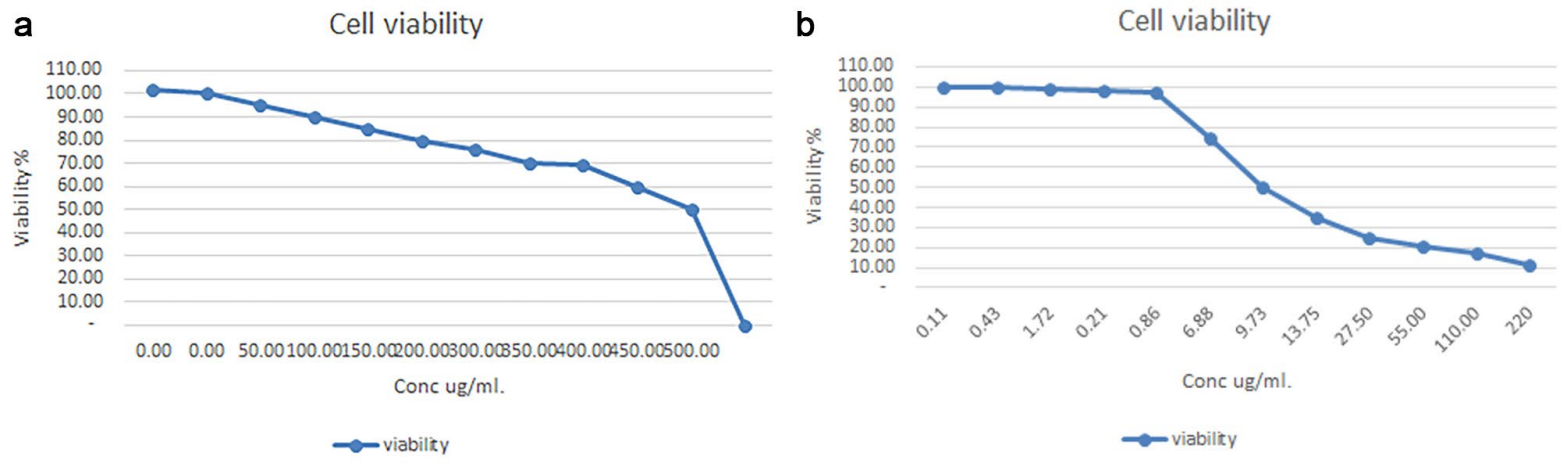
Evaluation of cell viability with serially papya seeds extract concentration using MTT assay. (**a**) C-166 cells. (**b**) Caco-2 cells

**Fig. 3:**
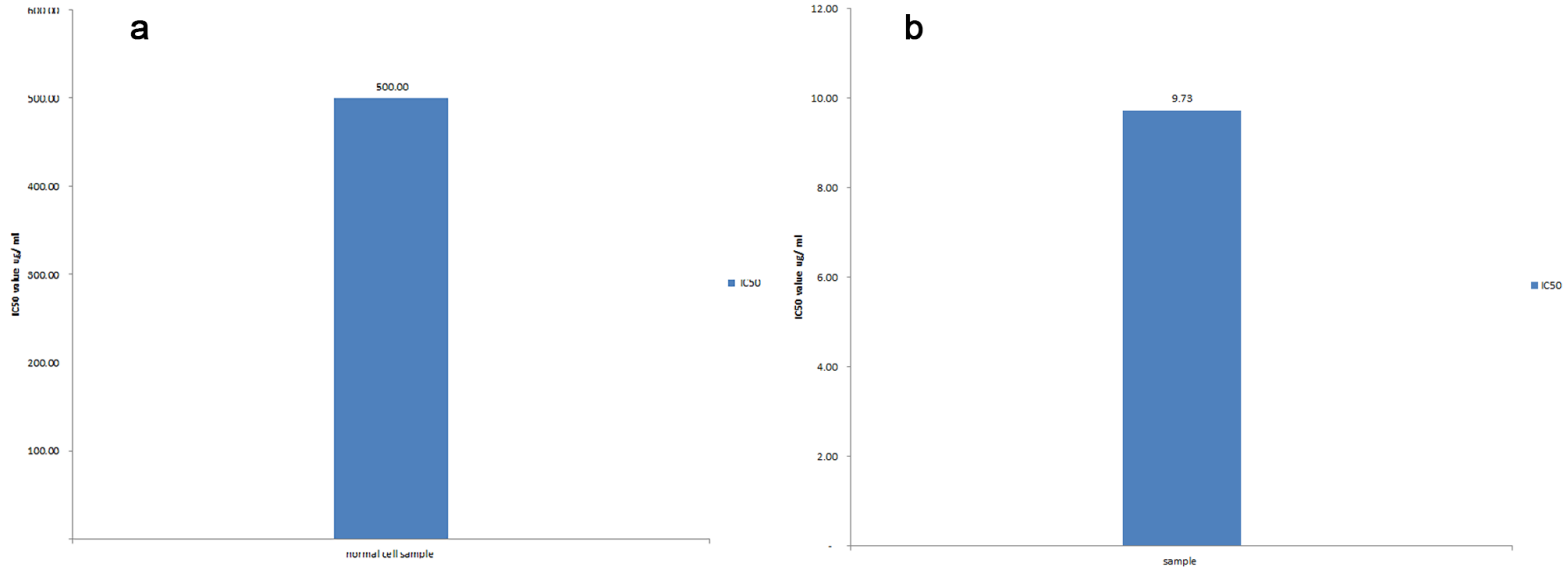
Evaluation of 50% inhibitory concentration (IC50) using Masterplex-2010 software. (**a**) C-166 cells. (**b**) Caco-2 cells

After 24 h of papaya seed extract treatment, some morphological changes were seen. At low dilutions, the cells appeared polygonal or spindle-shaped, with well-defined borders. However, by increasing extract concentrations cells shrunk and became void and irregular with condensed cell components (Fig. 1a,b). The IC_50_ value for the HTB-37Caco-2 cell line was 9.734 µg/mL and the IC_50_ value for normal cell line (C-166) was at 500 µg/mL (Figs. 2 and 3).

### The cell cycle analysis and mRNA expression levels of apoptosis-related genes

**Fig. 4:**
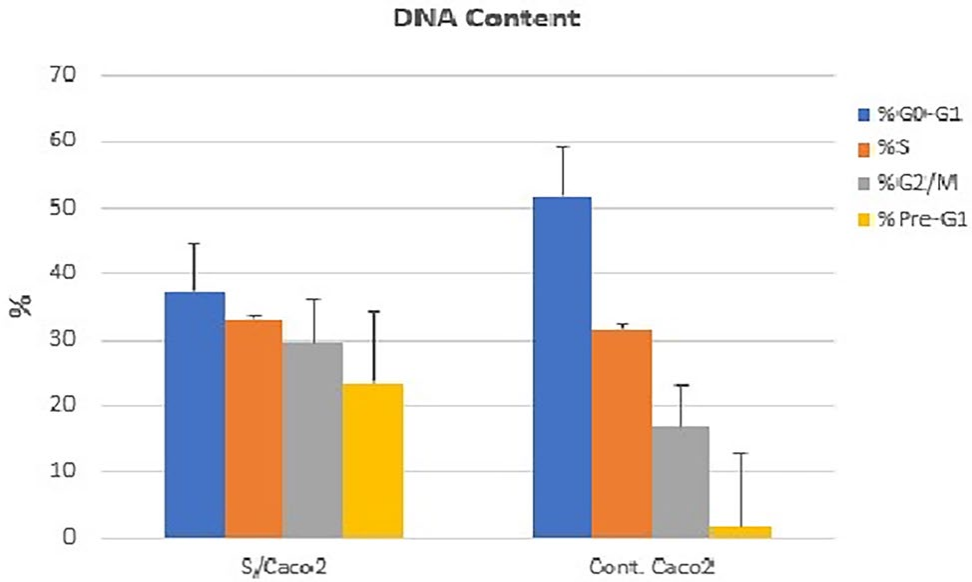
Effect of papaya seed extract on the cell cycle progression in colon cancer cells (Caco-2). The cell cycle distribution was analyzed by flow cytometry, which indicates that cell cycle arrest occurred in the G2/M phase. The treatment resulted in an increase in the percentage of apoptotic cells

**Fig. 5:**
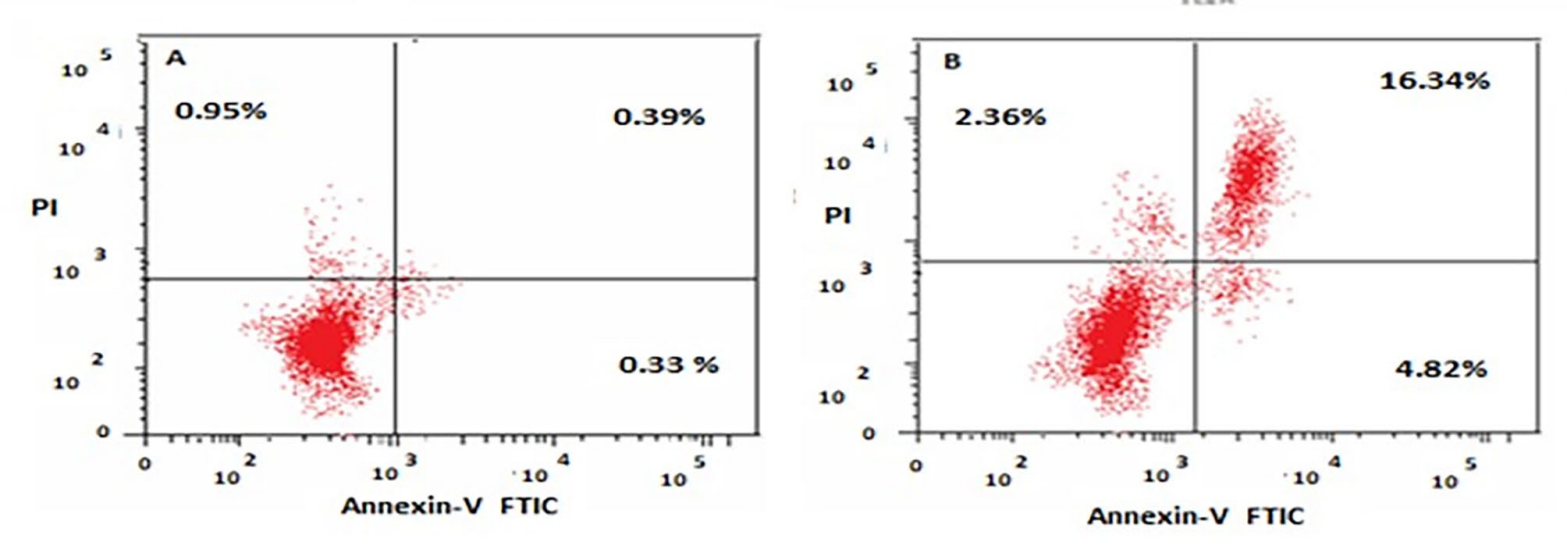
Evaluation of cell cycle profile using PI and annexin-V stains post Caco-2 cells treatment with papaya seed aqueous extract using flow cytometry. (**a**) Caco control cells. (b) Caco treated cell with IC50 papaya seed extract

Data revealed that there was an apoptotic rate of treated cells recording 23.52% for pre-G1, 37.26% for G1/G0, 33.08% for S, and 29.66% for G2/M phases, compared with the control cells which recorded 1.67% for pre-G1, 51.74% for G1/G0, 31.59% for S, and 16.67% G2/M phases, respectively. The data revealed that papaya seed extract induced a significant DNA accumulation at the G2/M phase Caco-2 cell arrest (Figs. 4 and 5).

**Fig. 6:**
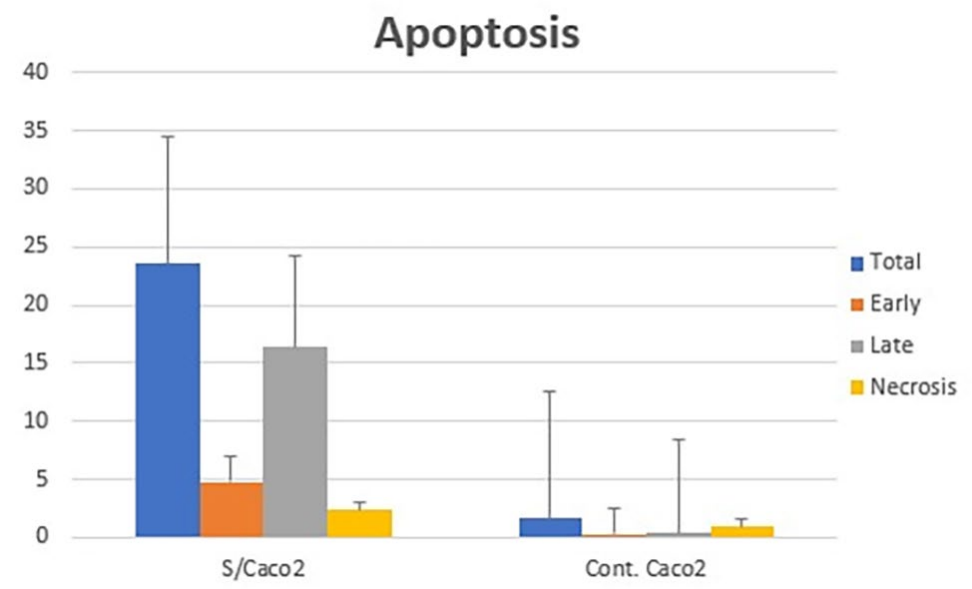
Cell cycle pattern analysis of papaya seed extract post -treated cells compared to control cells. The number of cells in each phase was counted according to the resulting fluorescence detected by flow cytometry analysis

**Fig. 7:**
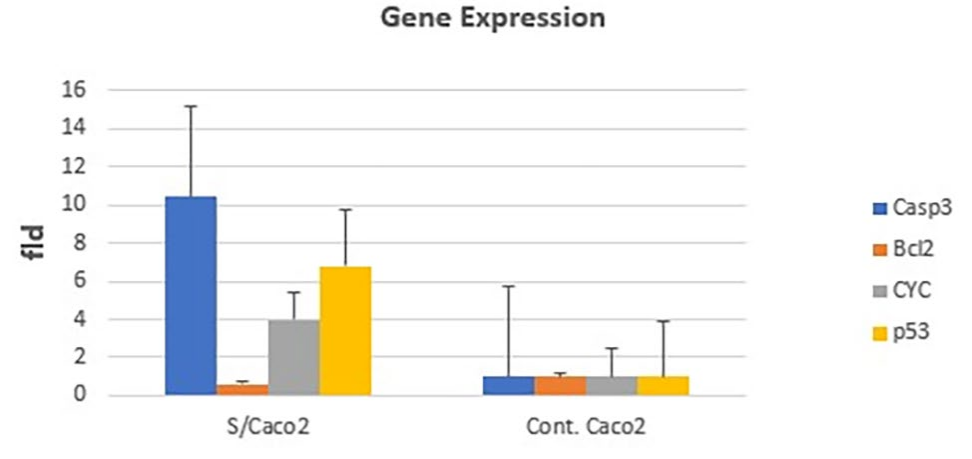
Gene expression level of *Casp-3, CyC*s, *Bcl-2*, and *p53* in colon cancer cells (Caco-2), using real-time PCR. The papaya seed extract-treated cells exhibited a significant increase in *Casp*-3, *p53*, and *CyC*s expression levels. In contrast, the down-regulation of the antiapoptotic *Bcl-2* gene expression level in colon Caco-2 cancer cell lines demonstrates the effectiveness of papaya seed extract in inducing apoptosis in the colon cell line. The values were considered to be statistically significant at (P < 0.05)

According to the data, early and late apoptosis were elevated compared with their levels in untreated control cells, and necrotic cells were also significantly increased (*P < 0.05*) throughout this time (Fig. 6). mRNA expression levels of apoptosis-related genes demonstrated that the treatment with papaya seed extract increased the expression of pro-apoptotic genes (*p53*, *CyCs*, and *Casp-3*) and decreased the expression of anti-apoptotic genes, explaining the induction of programmed cell death in cancer cells (Fig. 7).

## Discussion

HTB-37 Caco-2, and the healthy C-166 cell line were all used in the study to examine the effects of SE. The findings demonstrated that Caco-2 cells underwent considerable morphological changes after 24 h of treatment, including abnormalities, shrinkage, and compacted cell components. In contrast, normal cells were unaffected according to [20] angiogenesis is induced by cancer cells, cell proliferation is suppressed, and differentiation is impeded.

SE has the potential to be less hazardous to normal cells while still having cytotoxic effects on cancer cells, such as the Caco-2 cell line. Our results also demonstrate that papaya seed extracts with IC_50_ values of 9.734 µg/mL have a powerful cytotoxic activity, Similarly [9] who studied the cytotoxic effect of papaya seed aqueous extract on non-cancerous undifferentiated 3T3L1 fibroblasts, where both black and white seeds were evaluated. White seed extract inhibited cell proliferation by 50%, while black seed extract inhibited cell growth by 25%. These findings are consistent with our findings which showed that the IC_50_ of normal cells is 500ug/ml. Normal cells are generally less receptive to medications or chemicals than cancer cells; nevertheless, genetic mutations or other modifications make cancer cells more susceptible to specific therapies. As a result, the IC_50_ value of a drug or compound on a normal cell line may be greater than the IC_50_ value of the same substance or compound on a cancer cell line [21–23].

Also, García-Solís et al. [22]. noted that MCF-7 cell proliferation decreased after 72 h of treatment with papaya pulp. While Anilkumar et al. [6]. established that papaya extract is a powerful inhibitor of cell viability and migration in HepG2 cell lines with a half maximum inhibitory concentration (IC_50_) of 24.35 µg/mL and produces apoptotic alterations by down-regulating the *Bcl-2* and up-regulating *p53* and *Caspase-3* genes. Additionally, [23] reported that papaya seed extract have antitumor effects on several types of cancer cells, including PC-3 prostate cancer cells and acute promyelocytic leukemia HL-60 cells.

A study performed on the various stages of papaya ripening time found that ribosome-inactivating proteins (RIPs) isolated from *Carica papaya* leaves have cytotoxic activity in breast cancer cell line with the IC_50_ of 2.8 mg/mL, where the expression level of Bcl-2 protein decreased by the ratio 63%, and *p53* increased by 59.4% [24].

Some anticancer and DNA damage agents are known to work by arresting the cell cycle at various stages and then inducing apoptosis in cancer cells. Our results confirmed that the papaya seed extract induces cytotoxicity and apoptosis in the colon (Caco-2) cancer cell line. The cell cycle checkpoints are the pathways that enhance the cell death post exposure for toxins and determine the mechanism by which these pathways are regulated. According to Liao [25], who concurred with our findings, the anti-proliferative therapy involves causing G2/M phase arrest via a modulation mechanism, and mitochondria-mediated apoptosis in concentration is crucial for controlling cell cycle progression in breast cancer cells and Pre-G1 phase (apoptotic cells) were dramatically enhanced after cells were exposed to IC_50_ papaya seeds extracts causing G1-phase cell cycle arrest cycle advancement giving an opportunity for cells to perform repair mechanisms or follow the apoptotic pathway and these results agreed with Murad et al. findings [26]. Moreover Pre-G1 cell proliferation suggested an increase in apoptosis, which was accompanied by the creation of ROS and DNA damage and the subsequent activation of DNA repair mechanisms [27]. According to Somanah et al. [28], cancer colony formation decreases rapidly after exposure to papaya seed cell cycle due to G2/M phase arrest and expression of cell cycle genes interfered at S-phase checkpoint protein.

According to our findings, papaya seed extract increases mRNA expression of pro-apoptotic *Caspase-3* and *p53* genes while decreasing mRNA expression of the anti-apoptotic marker *Bcl-2* gene. *p53* activation triggers mitochondrial *cytochrome C*, which is followed by *Caspase-9* and *Caspase-3* activation. This is consistent with the findings of Singh et al. [29], who confirmed the increase of *p53* gene expression fivefold after being treated with papaya seed extract and down-regulation of the *Bcl-2* gene. Additionally, Hussar et al. [30] stated that the development of the apoptosome, a protein complex contributes to the conversion of pro*-Caspase-9* into active *Caspase-9*, which then activates *procaspase-3* to accelerate apoptosis.

Apoptosis-inducing factor (AIF) and cytochrome c from the inter-membrane space can enter the cytosol through macropores made in the outer mitochondrial membrane attributed to the *Bcl-2* protein family through caspase-3 which are proteolytic enzymes that are involved in apoptosis and inflammation regulation which is considered constantly active death enzyme [31]. Moreover, the nuclear transcription factor *p53* promotes apoptosis by causing cell cycle arrest and killing cells with significant DNA damage by inhibiting the transfer of damaged DNA to daughter cells via trans-activating some target genes [32].

Upregulation of the *Bcl-2* antiapoptotic proteins is a frequent method by which cancer cells can resist apoptosis since *the Bcl-2* gene has a crucial role in controlling apoptosis in the initiation, progression, and resistance to targeted therapy of tumor [33]. Unripe papaya seed aqueous extract,, exhibits renal protective efficacy up to 750 mg/kg in rats without causing renal impairment or oxidative stress because of a variety of beneficial compounds [34]. However, the total lethality of ripe papaya seed extract to fish at a dosage of 8 mg/ml has been claimed, according to [35]. The coat and oil of papaya seeds contain adequate antioxidant capabilities, adding credence to their potential nutritional and health benefits. As a result, papaya seeds are a one-of-a-kind medication that is safe for human ingestion [36]. This report was the first to demonstrate the effects of papaya seed aqueous extract on colon cancer cells, specifically highlighting gene expression and apoptotic profile analyses. Only a handful of studies employ various papaya fruit parts to treat colon cancer cell lines. One of these investigations confirmed that papaya seed extracts’ selectivity for colon cells as an anti-tumor on the DLD-1 (colon cancer cell line) and estimated the vitality of 20,000 cells per well after four days of incubation [37].

## Conclusion

The present study concluded that Papaya black seeds have a major role in the treatment of colorectal cancer. The novel aspect of our research is that colon cancer cells treated with papaya seed extract up-regulate the expression of *Caspase3, Cyc*s, *and p53*, triggering cell cycle arrest, apoptosis, and inhibition of colon cancer cells’ growth and proliferation. This method of treating colon cancer deserves more clinical research. To establish and comprehend the phytochemicals and pharmacokinetic effects of papaya seeds effects in the therapy of cancer.

### Limitation of the study

However, the expression of some relevant proteins using a western blot is recommended and GCMS for the extract should be performed to show the specific sorts of active phytochemicals that the Carica papaya seed extract employs to treat colon cancer.

## Data Availability

All data generated or analyzed during this study are included in this article.

## Abbreviations

HTB-37 Caco-2: Human colorectal adenocarcinoma cell line
Caco-2: Colorectal cancer cell line
C-166: Normal cell line mouse endothelial cell
CRC: Colorectal cancer
NSAIDs: Non-steroidal anti-inflammatory medicines
Apaf-1: Apoptotic protease activating factor
SE: *Carica Papaya Linn.* seeds aqueous extract
MTT: 3-(4,5-dimethylthiazol-2-yl)-2,5-diphenyltetrazolium bromide
IC_50_: Inhibitory concentration at 50%
EDTA: Diamine tetra acetic acid
PBS: Phosphate-buffered-saline
DMSO: Dimethyl sulfoxide
RIPs: Ribosome-inactivating proteins
AIF: Apoptosis-inducing factor
